# Extended‐spectrum beta‐lactamase‐producing *Escherichia coli* in common vampire bats *Desmodus rotundus* and livestock in Peru

**DOI:** 10.1111/zph.12456

**Published:** 2018-03-25

**Authors:** J. A. Benavides, C. Shiva, M. Virhuez, C. Tello, A. Appelgren, J. Vendrell, J. Solassol, S. Godreuil, D. G. Streicker

**Affiliations:** ^1^ Institute of Biodiversity, Animal Health and Comparative Medicine University of Glasgow Glasgow UK; ^2^ Faculty of Veterinary Medicine and Zootechnics University Cayetano Heredia of Peru Lima Peru; ^3^ Association for the Conservation and Development of Natural Resources Lima Peru; ^4^ Yunkawasi Lima Peru; ^5^ Laboratoire de Bactériologie Centre Hospitalier Universitaire (CHU) de Montpellier Montpellier France; ^6^ MIVEGEC (Laboratoire Maladies Infectieuses et Vecteurs, Ecologie, Génétique, Evolution et Contrôle) UMR CNRS 5290/IRD 224 Université Montpellier Montpellier France; ^7^ Department of Pathology Arnaud de Villeneuve Hospital CHU Montpellier Montpellier France; ^8^ INSERM U 1058 Montpellier France; ^9^ MRC‐University of Glasgow Centre for Virus Research Glasgow UK

**Keywords:** antibiotic resistance, *Desmodus rotundus*, ESBL‐producing *Escherichia coli*, multilocus sequence typing, Peru, plasmid typing

## Abstract

Antibiotic resistance mediated by bacterial production of extended‐spectrum beta‐lactamase (ESBL) is a global threat to public health. ESBL resistance is most commonly hospital‐acquired; however, infections acquired outside of hospital settings have raised concerns over the role of livestock and wildlife in the zoonotic spread of ESBL‐producing bacteria. Only limited data are available on the circulation of ESBL‐producing bacteria in animals. Here, we report ESBL‐producing *Escherichia coli* in wild common vampire bats *Desmodus rotundus* and livestock near Lima, Peru. Molecular analyses revealed that most of this resistance resulted from the expression of *bla*_CTX‐M‐15_ genes carried by plasmids, which are disseminating worldwide in hospital settings and have also been observed in healthy children of Peru. Multilocus sequence typing showed a diverse pool of *E. coli* strains carrying this resistance that were not always host species‐specific, suggesting sharing of strains between species or infection from a common source. This study shows widespread ESBL resistance in wild and domestic animals, supporting animal communities as a potential source of resistance. Future work is needed to elucidate the role of bats in the dissemination of antibiotic‐resistant strains of public health importance and to understand the origin of the observed resistance.

**Impacts**

- Extended‐spectrum beta‐lactamase (ESBL) resistance is a global threat to public health, and community‐acquired infections are increasingly reported.
- There are limited data on the role of wildlife and livestock in the circulation of ESBL‐producing bacteria, particularly in Latin America.
- The presence of ESBL‐producing *Escherichia coli* in both common vampire bats and livestock reveals a poorly understood and potentially zoonotic transmission cycle of ESBL resistance.

## 1 INTRODUCTION

Antimicrobial‐resistant (AMR) bacteria are responsible for hundreds of thousands of fatalities annually (World Health Organization, 2014). The majority of this problem is attributed to the spread of extended‐spectrum beta‐lactamase (ESBL)‐producing *Escherichia coli* and *Klebsiella pneumonia* (World Health Organization, 2014). Although most cases of ESBL‐producing bacteria are reported in clinical settings, increasing numbers of infections acquired outside of hospital settings (i.e., community‐acquired infections) have suggested that livestock and wildlife may form a zoonotic reservoir of ESBL for people living in close contact with animals (Guenther, Ewers, & Wieler, 2011). Supporting this hypothesis, ESBL‐producing *E. coli* have been found in several wild animals in Europe (Guenther et al., 2011); however, relatively few studies have been carried out on wild animals in low‐income countries (Carrillo‐Del Valle et al., 2016; Cristóbal‐Azkarate, Dunn, Day, & Amábile‐Cuevas, 2014; Guenther et al., 2011; Hasan et al., 2016; Liakopoulos et al., 2016), where the consequences of ESBL resistance can be exacerbated by a higher number of bacterial infections and more limited access to health facilities providing appropriate antibiotic treatment (Sosa et al., 2010).

While the role of bats as reservoirs of viruses is widely appreciated, bacterial infections have received less attention (Mühldorfer, 2013). Given their nightly feeding on antibiotic‐treated livestock and humans and widespread presence across Latin America, the common vampire bat, *Desmodus rotundus*, has a high risk of exposure to these bacteria and could potentially form a reservoir for transmission to other species. Although several recent studies have begun to characterize the diversity of pathogenic and non‐pathogenic bacteria in vampire bats (Adesiyun, Stewart‐Johnson, & Thompson, 2009; Bai et al., 2012; Carrillo‐Araujo et al., 2015; Chaverri, 2006; Zetun, Hoffmann, Silva, Souza, & Langoni, 2009), no study has evaluated this species for ESBL‐producing bacteria or investigated bacterial sharing with their livestock prey.

The aim of this study was to screen vampire bats and their surrounding livestock prey for ESBL‐producing Enterobacteriaceae in peri‐urban farms around Lima, and identify the bacterial strains and genes responsible for the observed phenotypic resistance to better understand the epidemiology of resistance in these communities. We report for the first time the presence of ESBL‐producing *E. coli* in vampire bats and livestock in Peru, suggesting a wide dissemination of AMR bacteria in the community.

## 2 METHODS

**Figure 1:**
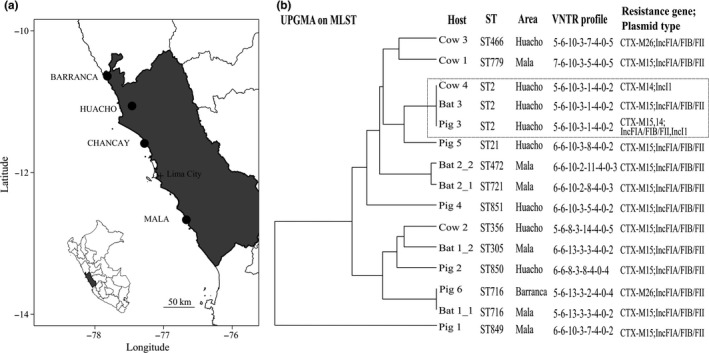
Map of the study area and phylogenetic relationships between ESBL‐producing bacteria from vampire bats and livestock in the Lima Region, Peru (a) Map with the location of the four vampire bat colonies sampled (b) Genetic relationship between isolates obtained by a Unweighted Pair Group Method with Arithmetic Mean (UPGMA) hierarchical clustering using the multilocus sequence typing (MLST) of loci dinB, icdA, pabB, polB, putP, trpA, trpB and uidA. The VNTR profile shown corresponds to the number of repeats from loci CVN001, CVN003, CVN004, CVN007, CVN014, CVN015, CVN016 and CCR001. The grey rectangle highlights isolates from three animals in Huacho showing the same genetic background. Isolates from the same individual are differentiated using an underscore

In October 2015, we collected faecal swabs from 81 vampire bats in four colonies located in four districts (*N* = 29 in Mala district, 20 in Barranca, 20 in Huacho and 12 in Chancay) of the Lima Region of Peru (Figure 1a). We also collected fresh faecal swabs from 20 cows, eight pigs, five sheep, two horses and two donkeys from farms located <5 km from bat colonies, an area within the typical foraging range of vampire bats (Trajano, 1996). Samples were collected from six small‐scale farms that kept <30 animals each. Questionnaires to farmers and visual inspections of bite wounds confirmed that bats fed in all farms in the month prior to sampling and that farmers commonly treated their animals with tetracycline and penicillin. Twenty‐five per cent of samples came from livestock with fresh bites. Swabs from both bats and livestock were first screened for ESBL‐producing Enterobacteriaceae by direct incubation (on average, <1 hr after collection) at 37°C for 24 hr in ChromID ESBL (bioMérieux, Marcy l'Etoile, France). Samples exhibiting bacterial growth were subcultured to confirm growth after 24 and 48 hr of incubation. We used matrix‐assisted laser desorption/ionization–time‐of‐flight (MALDI‐TOF) mass spectrometry (Bruker Daltonics, Bremen, Germany) to identify the species of ESBL‐positive samples. From the 58 ESBL‐positive samples, 15 were *E. coli* (five from bats and 10 from livestock). The remaining 43 isolates included non‐Enterobacteriaceae Gram‐negative bacilli (e.g., *Pseudomonas* spp. and *Acromobacter* spp.). Susceptibility testing to individual β‐lactam antibiotics was performed using the disc diffusion method on Mueller–Hinton agar (Matuschek, Brown, & Kahlmeter, 2014) and was interpreted according to the European Committee on Antimicrobial Susceptibility Testing (EUCAST) clinical breakpoints (version 5.0). ESBL production was confirmed with the double‐disc synergy test.

We assessed the genetic background of the ESBL‐producing *E. coli* by performing multilocus sequence typing (MLST) (http://bigsdb.web.pasteur.fr/) and variable number of tandem repeats (VNTR) (Lindstedt, Brandal, Aas, Vardund, & Kapperud, 2007). The genetic basis of the observed resistance was identified using multiplex PCR and DNA sequencing targeting the most prevalent ESBL‐encoding genes (e.g., *bla*_CTX‐M_, *bla*_TEM_ *, bla*_SHV_ and *bla*_OXA‐1_‐like genes) (Dallenne, Da Costa, Decré, Favier, & Arlet, 2010). Plasmids carrying resistant genes were identified and typed using the PCR‐based replicon typing method and the plasmid relaxase gene typing method (Carattoli et al., 2005; Compain et al., 2014).We also evaluated the ability of plasmids to conjugate using mating experiments with the azide‐resistant *E. coli* strain J53 as a recipient.

## 3 RESULTS

We found 15 *E. coli* isolates that were intermediate or resistant to most β‐lactam antibiotics, except imipenem and ertapenem, in both vampire bats and livestock (Table S1). Molecular analysis revealed the presence of *bla*_CTX‐M‐15_ resistance gene alone in 11 isolates (all five bat and six livestock isolates including two cows and four pigs) and associated with *bla*_CTX‐M‐14_ in one isolate (“Pig 3,” Figure 1b). The *bla*_CTX‐M‐26_ and *bla*_CTX‐M‐14_ genes were found in two (“Cow 3” and “Pig 6”) and one (“Cow 4”) isolates of livestock, respectively. The *bla*_CTX‐M15_ and *bla*_CTX‐M26_ genes were associated with IncFIA/FIB/FII plasmid, and the *bla*_CTX‐M14_ was associated with IncI1 plasmid. Mating experiments showed that these plasmids were able to conjugate, suggesting their potential circulation between different bacterial strains.

Strain genotyping by MLST revealed high diversity among *E. coli* isolates across hosts and sampling locations: bat isolates belonged to sequence types (STs) ST2, ST305, ST472, ST716 and ST721, whereas livestock isolates belonged to ST2, ST21, ST356, ST466, ST479, ST716 and 3 novel STs registered as ST849, ST850 and ST851. All isolates had unique VNTR haplotypes, except for ST2, for which isolates from a bat, a cow and a pig in Huacho had the same haplotype.

## 4 DISCUSSION

Extended‐spectrum beta‐lactamase‐producing *E. coli* carrying *bla*_CTX‐M‐15_ genes have disseminated worldwide, causing a large number of nosocomial and community‐acquired urinary tract and bloodstream infections among humans (Guenther et al., 2011). *bla*_CTX‐M‐15_ and *bla*_CTX‐M‐14_ genes were reported in healthy children in Peru and Bolivia, and IncFIA/FIB/FII and IncI1 plasmids have been suggested to play a major role in the dissemination of these genes across humans in Latin America (Pallecchi et al., 2007). In our study, *bla*_CTX‐M_ genes were carried by these same broad‐host‐range transmissible plasmids, suggesting that wildlife and livestock may also disseminate this ESBL resistance. The high diversity of *E. coli* STs found here, which do not overlap with clones found in wild birds of Chile or Argentina (Báez et al., 2015; Liakopoulos et al., 2016), suggests dissemination of these plasmids between different bacterial strains.

A pig and the bat carried the same *bla*_CTX‐M‐15_ gene within the same plasmid and the same *E. coli* ST, suggesting ESBL‐producing bacterial exchange between bats and livestock or contamination from a common source such as human sewage. Several routes of bacterial transmission between bats and livestock are possible. For example, faecal–oral transmission from livestock to bats could occur if bats ingest contaminated faecal material present on livestock skin or in the environment. Environmental contamination of bats with humans or livestock bacteria can increase if human or livestock sewage is used in farms. Alternatively, transmission is possible during blood feeding or contact with the wound, although this should be less frequent for *E. coli*, given that this bacterium is not commonly found in the blood of healthy animals. Finally, livestock and humans could also be exposed to bat bacteria through guano, as bat roosts in this area are mainly located in tunnels that cover parts of irrigation systems for crops.

The role of wildlife in the dissemination of resistant genes is supported by the recent isolation of ESBL‐producing bacteria carrying *bla*_CTX‐M‐15_ genes in wild birds of Nicaragua, Chile and Argentina, and raises public health concerns given the implications of these bacteria in both nosocomial and community‐acquired infections in humans (Guenther et al., 2011; Hasan et al., 2016; Liakopoulos et al., 2016). Our findings highlight the need to elucidate the role of bats and livestock in AMR bacterial transmission cycles and what risks these community sources may pose for zoonotic transmission to humans. Specifically, future work should investigate whether bat populations are capable of maintaining ESBL‐producing bacteria or whether these resistant bacteria result from frequent exchanges with livestock or humans through the environment (e.g., water and soil).
